# Vanadium-Doped Hafnium Oxide: A High-Endurance Ferroelectric Thin Film with Demonstrated Negative Capacitance

**DOI:** 10.1021/acs.nanolett.4c05671

**Published:** 2025-02-07

**Authors:** Ehsan Ansari, Niccolò Martinolli, Emeric Hartmann, Anna Varini, Igor Stolichnov, Adrian Mihai Ionescu

**Affiliations:** † Nanoelectronic Device Laboratory, EPFL, Lausanne 1015, Switzerland; ‡ ENS Paris-Saclay, Gif-sur-Yvette 91190, France

**Keywords:** vanadium-doped hafnium oxide (V:HfO_2_), ferroelectric thin film, high endurance, CMOS-compatible, atomic layer deposition (ALD), negative capacitance

## Abstract

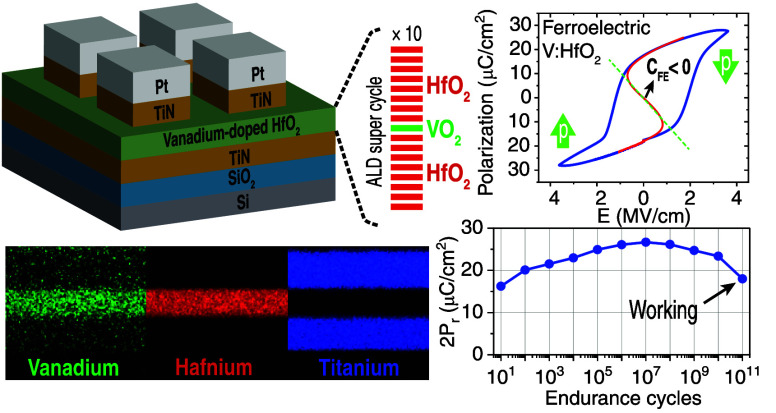

This study proposes and validates a novel CMOS-compatible ferroelectric thin-film insulator made of vanadium-doped hafnium oxide (V:HfO_2_) by using an optimized atomic layer deposition (ALD) process. Comparative electrical performance analysis of metal–ferroelectric–metal capacitors with varying V-doping concentrations, along with advanced material characterizations, confirmed the ferroelectric behavior and reliability of V:HfO_2_. With remnant polarization (*P*_r_) values up to 20 μC/cm^2^, a coercive field (*E*_c_) of 1.5 MV/cm, excellent endurance (>10^11^ cycles without failure, extrapolated to 10^12^ cycles), projected 10-year nonvolatile retention (>100 days measured), and large grain sizes of ∼180 nm, V:HfO_2_ emerges as a promising robust candidate for nonvolatile memory and neuromorphic applications. Importantly, negative capacitance (NC) effects were observed and analyzed in V:HfO_2_ through pulsed measurements, demonstrating its potential for NC applications. Finally, this novel ferroelectric shows potential as a gating insulator for future 3-terminal vanadium dioxide Mott-insulator devices and sensors, achieved through an all-ALD process.

Research on CMOS-compatible ferroelectric thin films has intensified since the discovery of ferroelectricity in silicon-doped hafnium oxide (Si:HfO_2_) by Böscke et al.^1^ Lead-free HfO_2_-based ferroelectrics have been shown to be among the CMOS-compatible ferroelectrics with the highest remnant polarization (*P*_r_) and high scalability. Applications span from nonvolatile (NV) memories (such as ferroelectric RAM and multilevel cell memory) to steep-slope and negative capacitance (NC) devices (e.g., ferroelectric FET and NC-FET), as well as programmable gates and neuromorphic devices.^2−4^ Several possible dopants have been introduced for HfO_2_ ferroelectrics, including silicon (Si), zirconium (Zr), lanthanum (La), gadolinium (Gd), gallium (Ga), aluminum (Al), and yttrium (Y).^5−9,11^ Each of these dopants possesses specific characteristics; however, Si and Zr are generally regarded as the most promising due to their compatibility with CMOS technology. In this work, we investigate for the first time the ALD deposition, processing, and properties of vanadium-doped hafnium oxide (V:HfO_2_) and report its unique electrical and ferroelectric material characteristics, reliability, and optimization. This new ferroelectric thin film is compatible not only with CMOS technology but also with the gating of future VO_2_ phase-change switches and sensors, utilizing a simplified all-ALD process to deposit both VO_2_ and V:HfO_2_ within the same ALD process and with an identical vanadium precursor.

The proposed device vehicle for our investigation is a metal–ferroelectric–metal (MFM) capacitor, which allows a direct comparison of the main figures of merit with those of other high-*k* ferroelectrics. The process flow is depicted in Figure 1a, where a 19 nm back electrode of titanium nitride (TiN) was deposited by RF sputtering on top of a silicon wafer with 200 nm of silicon oxide. Then a V:HfO_2_ layer (16 nm) was deposited by ALD at 240 °C; tetrakis(ethylmethylamid)hafnium(IV) (TEMAH) and water (H_2_O) are used as HfO_2_ precursors, and tetrakis(ethylmethylamino)vanadium (TEMAV) and ozone (O_3_) are used for VO_2_. TEMAV is one of the most common precursors for VO_2_ ALD;^12^ therefore, it can be utilized to deposit both VO_2_ and V:HfO_2_ (as a ferroelectric gate material for 3-terminal Mott insulator devices) in a single ALD process. To optimize the V concentration, eight sets of cycle sequences have been tested to deposit V:HfO_2_ at different VO_2_ cycle ratios from 3% to 11.1%. VO_2_ doping cycles were distributed homogeneously between HfO_2_ cycles, and all the ALD processes were performed at 240 °C. The structure of ALD cycles for an optimal concentration of 5.9% for VO_2_ (Hf:V cycle ratio of 16:1) is illustrated in Figure 1b. A 19 nm TiN top electrode was then deposited by RF sputtering, and the entire stack was annealed in nitrogen (N_2_) atmosphere at 600 °C for 2 min using a rapid thermal processing (RTP) furnace. Finally, the MFM capacitors were fabricated on diced dies by RF sputtering of 50 nm of platinum (Pt), photolithography (direct laser writing), and ion beam etching (IBE) of the unwanted Pt and top TiN. For further investigation, similar MFM structures were fabricated under various annealing temperatures ranging from 400 to 800 °C for 2 min and with different thicknesses of V:HfO_2_ layer, ranging from 8 to 24 nm. In addition, for NC tests, metal–ferroelectric–insulator–metal (MFIM) devices were made similarly with 3 nm of Al_2_O_3_ as a linear dielectric deposited with ALD before the V:HfO_2_ layer.

**Figure 1:**
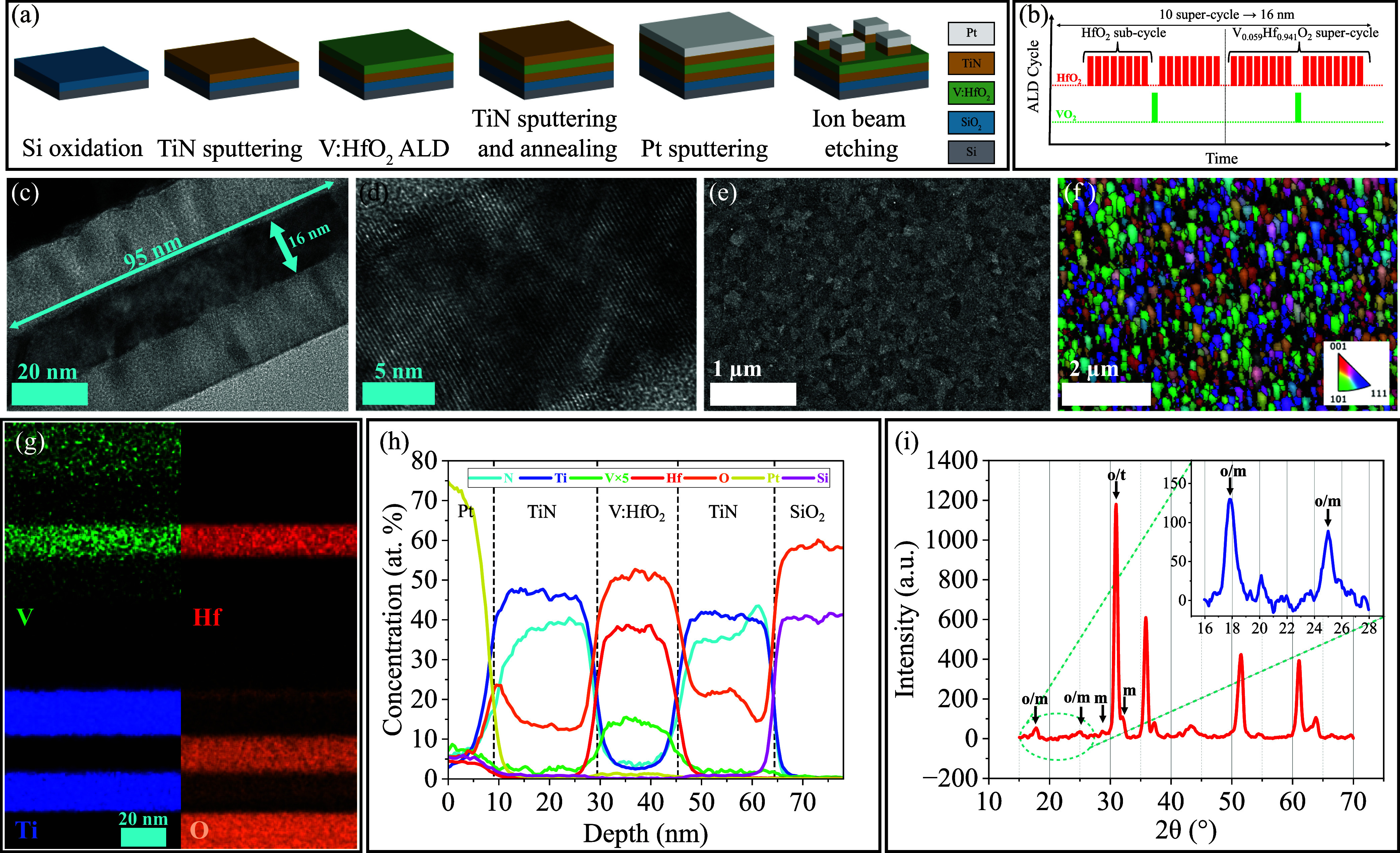
(a) Schematic and fabrication process flow of the MFM capacitors. (b) Schematic of the optimized ALD process and cycle structure for 5.9% ferroelectric V:HfO_2_. (c) TEM and (d) high-resolution TEM images of the cross section of TiN/V:HfO_2_/TiN MFM stack which was electrically woken up. Visible atomic fringes and large grains of >95 nm confirm the crystal quality. (e) Top SEM image of MFM structure after removal of the top TiN layer. Grain domains with a size of ∼200 nm are visible. (f) EBSD map of the same sample, showing an average grain size of ∼180 nm. (g) TEM EDX mapping and (h) elemental profile of the cross section of the MFM capacitor. In the ferroelectric layer, an atomic concentration of 5.6% was measured for V. (i) GXRD spectra of the annealed TiN/V:HfO_2_/TiN MFM stack. Inset represents low angle peaks with longer acquisition time and better precision.

The resulting capacitive V:HfO_2_ stack with optimum 5.9% V-doping level was characterized by different methods including transmission electron microscopy (TEM), energy dispersive X-ray spectroscopy (EDX), scanning electron microscopy (SEM), electron backscatter diffraction (EBSD), grazing-incidence X-ray diffraction (GIXRD), piezoresponse force microscopy (PFM), and X-ray photoelectron spectroscopy (XPS). TEM and EDX images and analyses were obtained from a lamella cross-sectional cut out of an electrically woken-up capacitor using focused ion beam technique. In the TEM cross-sectional image, a thickness of 16 nm is observed for 5.9% V:HfO_2_ with 600 °C annealing, as designed with the number of ALD cycles (Figure 1b). Figure 1c,d presents bright-field TEM images of atomic fringes in the stack, suggesting that relatively large grains of >95 nm with high crystal quality are present. Figure 1e depicts the SEM analysis of the surface, which was conducted after the removal of the top TiN layer of a 1.5 × 1.5 cm^2^ die by wet etching. In the figure, remarkably large grains, reaching sizes of ∼200 nm, are visible. In order to explore their uniformity and distribution of sizes and orientations, an EBSD analysis was performed (Figure 1f) on the same die. From this image obtained with a 30 nm step size, an equivalent circle diameter distribution has been extracted, suggesting an average grain size of ∼180 nm. Note that for the indexing of the diffraction data, the cubic phase of HfO_2_ was chosen. Finally, the orientation data have been used to plot pole figures of the {100}, {110}, and {111} family of planes, showing a very weak ⟨110⟩ texture in the *z*-direction. By comparing the results of the TEM, SEM, and EBSD analyses, it was confirmed that the average grain size of the V:HfO_2_ layer is approximately 180 nm. To the best of our knowledge, the maximum reported grain size of a HfO_2_ ferroelectric layer in an MFM structure with similar processes is ∼50–70 nm for Si-doped HfO_2_ and HZO.^13,14^ However, larger grains (∼230 nm) have been observed in Si-doped HfO_2_ only in the presence of an insulating layer (MFIM structure).^13^ This enhanced crystallization of V:HfO_2_ layer has not been reported in similarly fabricated HfO_2_ MFM structures with other dopants, underscoring the unique effect of vanadium doping and paving the way for the development of monocrystalline ferroelectric gated devices.

Additionally, EDX was used to map the elemental distribution of the MFM stack. The extracted elemental mapping images and concentration profile, depicted in Figure 1g,h, confirm the sharp boundaries of the layers and the elemental concentration of the V:HfO_2_ layer. A V-doping level of 5.6% was measured in the ferroelectric V:HfO_2_ layer by EDX, which closely matches the VO_2_ ratio of 5.9% in the preformed ALD process.

Figure 1i depicts the GIXRD spectrum which was acquired at an incidence angle of 0.45° using a monochromatic copper Kα beam from a whole 1.5 × 1.5 cm^2^ die of the MFM stack. In agreement with the literature,^1^ the most intense peak is attributed to either the (111) plane of the orthorhombic (*o*) phase of HfO_2_ or the (101) plane of the tetragonal (*t*) phase. Around the main peak, the minority presence of the monoclinic (*m*) phase is evidenced by the peaks corresponding from left to right to the (1̅11) and (111) planes. The relatively strong signal coming from the two weak peaks on the left (from left to right, (100)_*o*_ and (110)_*o*_, or (100)_*m*_ and (011)_*m*_) is a good indication in favor of the ferroelectric orthorhombic phase over the paraelectric tetragonal phase. This interval was also scanned with a longer acquisition time, as reported in the inset.

PFM analysis was performed on the MFM capacitors with an Asylum Research Cypher AFM instrument using conductive doped-diamond tips in a nitrogen gas atmosphere. An external Zurich Instrument HF2LI lock-in amplifier was used to read out the displacement amplitude and phase value of the PFM loops and image. Figure 2a-c,e-g shows the DC off-field PFM mapping within an area of 1 μm^2^. Switchable piezoelectric domains with lateral sizes of up to 200–300 nm are visible, which aligns with other analyses and further confirms the presence of relatively large ferroelectric grains. Figure 2d,h represents the DC off-field and DC on-field PFM displacement and phase loops of a point marked with an arrow in Figure 2a. The DC on- and off-field curves are well overlapped (which is a sign of good retention), and significant piezoresponse is observed. A perfect 180° phase jump in Figure 2h further confirms the high-quality piezoresponse. Nonetheless, a slight shift in coercive fields toward the positive direction is observed, which could be attributed to the imprint effect. The schematic of the voltage waveform used to acquire the PFM loops is included in Figure S3 (Supporting Information).

**Figure 2:**
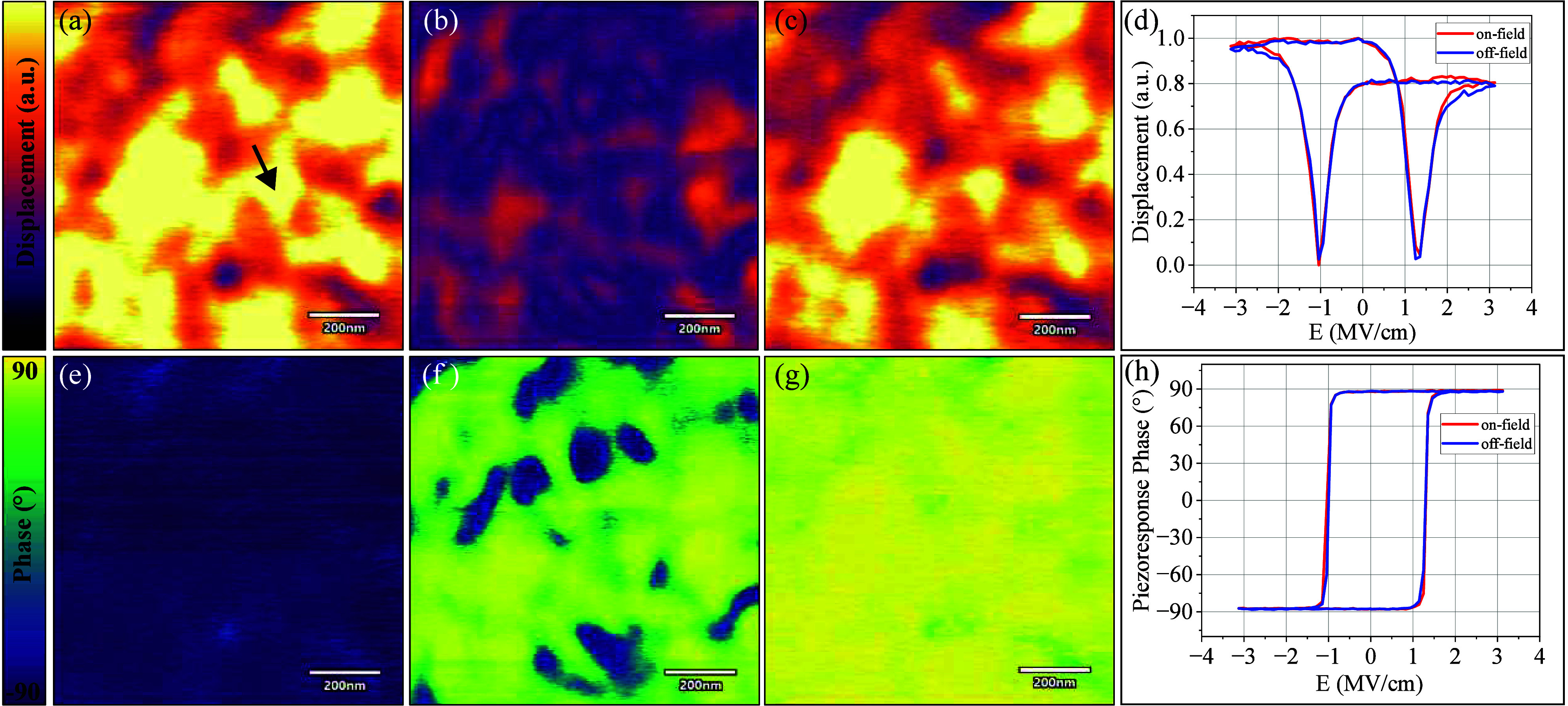
DC off-field PFM mapping images of amplitude and phase of local piezoresponses within same (1 μm^2^) area in (a and d) negatively poled state, (b and e) mixed state, and (c and f) positively poled state; prepolarization was performed using DC biases of −5, 2, and 5 V, respectively. The spatial uniformity of the phase and amplitude images over a 1 h scan time indicates robust retention of the ferroelectric. (d and h) DC off-field and DC on-field PFM loops of amplitude and phase of local piezoresponse measured at the marked point in panel a. These experiments were carried out on an MFM capacitor with a thickness of 16 nm and 5.9% V-doping level.

XPS analysis of V:HfO_2_ layers in MFM structures, following the removal of the top TiN layer (Figure S1), confirmed the dominant presence of V^3+^, along with a fraction of V^4+^ and a minor fraction of V^5+^. The ionic radii of these vanadium oxidation states fall between those of Si^4+^ and Zr^4+^, established HfO_2_ dopants known for enhancing ferroelectricity (Figure S4). This suggests that vanadium primarily acts as a substitutional cation dopant, similar to Si, Zr, Y, Al, and La,^15^ though further analysis is needed to confirm this hypothesis. Additionally, strain induced by doping is a critical factor in driving HfO_2_ toward ferroelectric phases.^1^ The optimum vanadium atomic concentration of 5.9% is positioned between ∼50% for Zr and ∼3% for Si,^6^ aligning with their respective ionic radii and the strain generated due to the radius difference relative to Hf. The copresence of multiple vanadium valences with varying ionic sizes makes vanadium a unique multivalent dopant capable of inducing ferroelectricity in HfO_2_. Further details on the mechanism of observed enhancements due to vanadium doping are provided in the Supporting Information.

Electrical characterization was performed on MFM capacitors with different V concentrations in their V:HfO_2_ layer by polarization–voltage (*P*–*V*) and capacitance–voltage (*C*–*V*) measurements at room temperature (RT). Before the *P*–*V* and *C*–*V* characteristics were obtained, a so-called wake-up procedure was carried out, by applying 500 initial bipolar cycles of 5 V, followed by 500 bipolar cycles of 6 V rectangular pulses at the same frequency. *P*–*V* hysteresis loops were measured by applying a 10 kHz triangular voltage waveform with an amplitude of 6 V, and the *C*–*V* measurement was done using a 100 kHz AC 30 mV RMS signal added to a DC voltage sweep of 6 V amplitude. The amplitude of ±6 V (±3.75 MV/cm) was chosen to achieve the maximum saturated *P*_r_ without causing ferroelectric breakdown. The measurements were performed on a Cascade Summit 200 or SUSS MicroTec PMC150 cryogenic probe station, using a Keithley 4200 parameter analyzer equipped with source-measurement unit (SMU), pulse-measurement unit (PMU) and capacitance–voltage unit (CVU). Given the size of the MFM capacitors (100 × 100 μm^2^) and the thickness of the V:HfO_2_ layer, remnant polarization–electric field (*P*_r_–*E*); current density–electric field; and, using the ideal parallel plate capacitor approximation, relative permittivity–electric field (ε_r_–*E*) loops were extracted from P–V and C–V measurements for different V concentrations, as depicted in Figure 3.

**Figure 3:**
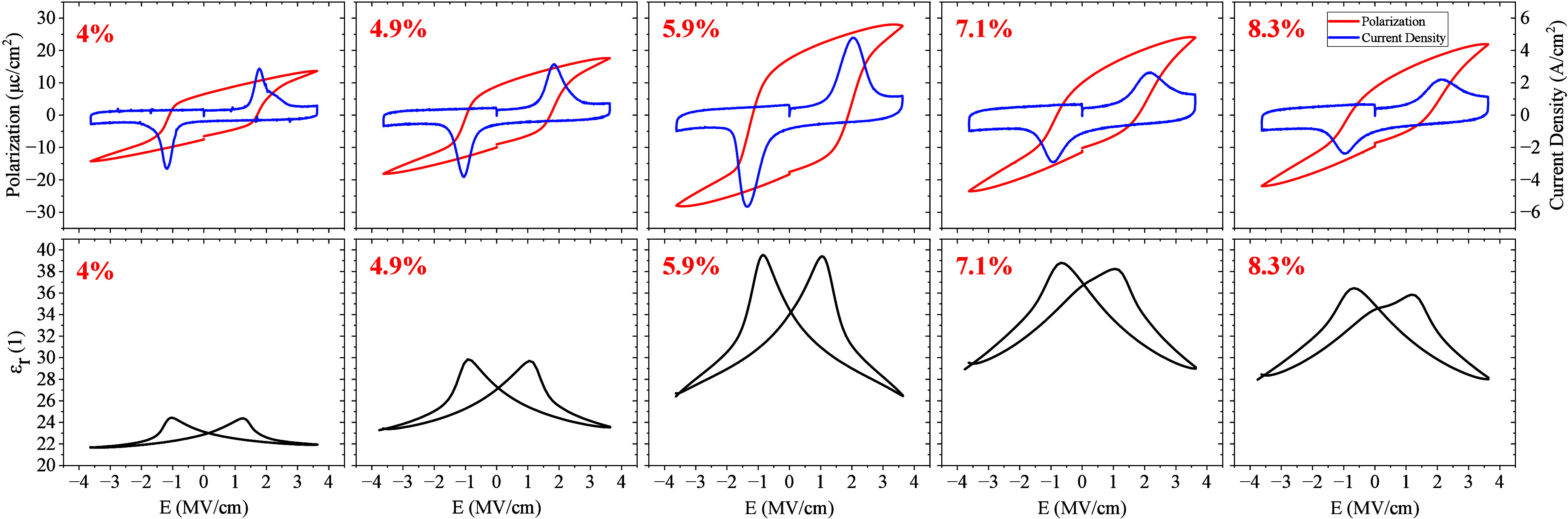
*P*_r_–*E* and current density–electric field loops for different VO_2_ ALD cycle ratios (top row), measured using a 6 V, 10 kHz triangular stimulation. ε_r_–*E* curves (bottom row) are measured by a CV measurement method with a 100 kHz AC 30 mV RMS signal added to a DC voltage sweep of 6 V amplitude. All the MFM capacitors were annealed at 600 °C for 2 min. The highest remnant polarization of 17 μC/cm^2^ and the highest ε_r_ change observed at a VO_2_ ALD cycle ratio of 5.9%, which is considered as the optimum fabrication condition.

For better comparison, *P*_r_ and ε_r_ were plotted as a function of VO_2_ ALD cycle ratio for eight different ratios in Figure 4a. Evidently, 5.9% V-doping had the largest *P*_r_ and the highest quality ferroelectric response. By making five similar batches of MFM capacitors with 5.9% V-doping, an average remnant polarization of 17 μC/cm^2^ was observed, with a maximum of 20 μC/cm^2^. In the ε_r_-VO_2_ ALD cycle ratio graph (Figure 4a) the ε_r_ values represent the minimum values of the ε_r_–*E* curves at maximum electric fields. It can be observed that as the V ratio increases from 3% to 5.9%, ε_r_ also increases and then approximately saturates at around 7%. A significant increase in ε_r_ occurs at 5.9% V-doping, with a ε_r_ of 26.4. This increasing trend is similar to what has been previously reported for hafnium zirconium oxide (HZO), where the increase in ε_r_ is attributed to a reduction in the monoclinic phase fraction.^7^ However, for V:HfO_2_, the increasing trend levels off after 7%, leading to saturation, which could be due to the stabilization of the minimum monoclinic phase fraction. Coercive field (*E*_c_), which is defined as *E*_c_ = (*E*_c+_ – *E*_c–_)/2, was also extracted from *P*_r_–*E* curves and plotted as a function of the VO_2_ ALD cycle ratio in Figure S5. A maximum *E*_c_ of 1.5 MV/cm was also observed for 5.9% V-doping.

**Figure 4:**
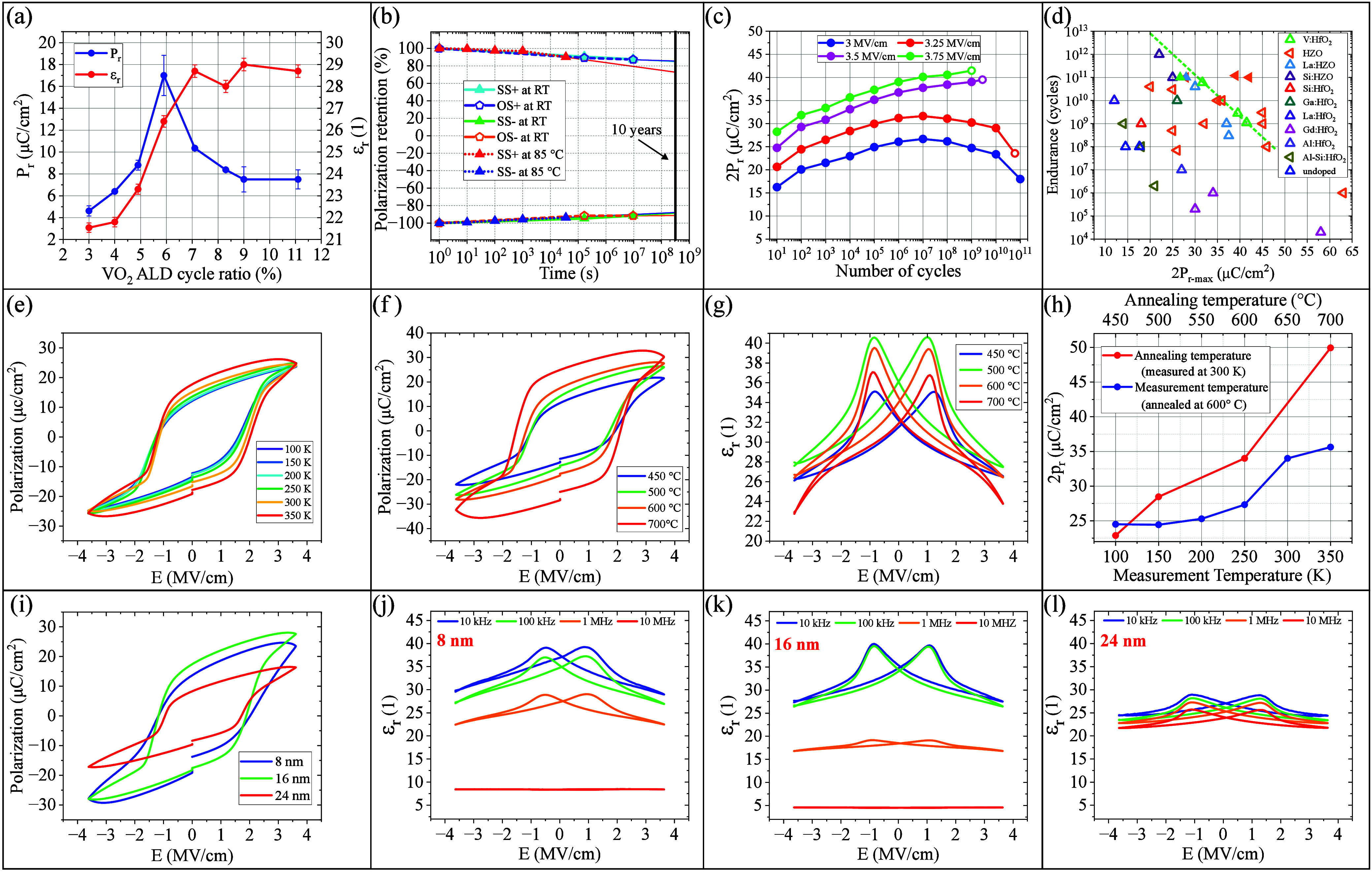
(a) *P*_r_ and ε_r_ at RT as a function of the VO_2_ ALD cycle ratio. The error bar represents the standard deviation of five similar batches. (b) SS± and OS± retention test of 5.9% V:HfO_2_ in MFM capacitor devices at 85 °C and at RT. (c) Endurance characteristic of an MFM capacitor with 5.9% V:HfO_2_ layer under 1 MHz, 4.8 V (3 MV/cm), 5.2 V (3.25 MV/cm), 5.6 V (3.5 MV/cm), and 6 V (3.75 MV/cm) pulse stimulation. An endurance of up to 10^11^ cycles without failure was observed under a pulse stimulation of 4.8 V. Empty symbols represent breakdown. (d) Benchmark study of endurance versus maximum 2*P*_r_ during the endurance test, comparing V:HfO_2_ and recent reports on HfO_2_-based ferroelectrics. Empty symbols represent breakdown. The data were obtained from the following references: HZO,^16−23^ La:HZO,^23,24^ Si:HZO,^25^ Si:HfO_2_,^26^ Ga:HfO_2_,^9^ La:HfO_2_,^8^ Gd:HfO_2_,^27^ Al:HfO_2_,^10^ Al:Si:HfO_2_,^28^ undoped HfO_2_.^29^ (e) *P*_r_–*E* hysteresis loops at different temperature conditions from 100 to 350 K, (f) *P*_r_–*E* and (g) ε_r_–*E* curves at different annealing temperatures, ranging from 450 to 700 °C. (h) 2*P*_r_ as a function of annealing and measurement temperatures, extracted from panels e and f. (i) *P*_r_–*E* curves and (j–l) ε_r_–*E* curves and frequency dispersion of 8, 12 and 24 nm V:HfO_2_ layers measured at RT.

The reliability characteristics of ferroelectric V:HfO_2_ were evaluated through polarization retention and cycling endurance tests. Same-state (SS) and opposite-state (OS) retention tests were performed using similar MFM capacitors with 50 μs read/write pulses of ±5 V in two sets of experiments at 85 °C and at RT. At 85 °C, as shown in Figure 4c, a retention of >90% was observed after 10 h in both polarization directions within same-state tests following positive (SS+) and negative (SS−) prepolarization. The capacitors were maintained at 85 °C throughout the electrical measurements and the delay time. At RT, after ∼110 days, polarization retentions of >87% was observed in SS± and OS± experiments. Long-term retention was extrapolated to 10 years, as illustrated in Figure 4, demonstrating the material’s outstanding retention characteristics.

For the endurance test, 1 MHz pulse stimulation of 4.8 V (3 MV/cm), 5.2 V (3.25 MV/cm), 5.6 V (3.5 MV/cm), and 6 V (3.75 MV/cm) were applied to similar MFM capacitor stacks with a size of 50 × 50 μm^2^. A positive-up-negative-down (PUND) method with 1 kHz triangular pulses of similar voltage amplitudes was employed to accurately measure the *P*_r_ value of the ferroelectric material and exclude leakage and dielectric current contributions. Figure 4c shows a robust endurance of up to 10^11^ cycles without failure with a final 2*P*_r_ value of 18 μC/cm^2^ under a pulse stimulation of 4.8 V. The mechanism of this improvement is further discussed in the Supporting Information. Extended endurance tests were not feasible due to the excessively long testing time. In general, remnant polarization increased by cycling until ∼10^7^ cycles due to wake-up effect; it then decreased due to the fatigue effect. The capacitors experienced failure when stimulated with pulse amplitudes larger than 4.8 V.

To broaden the scope of this investigation, a benchmark study was conducted to compare endurance cycles versus maximum 2*P*_r_ values (observed during endurance tests) across leading reported HfO_2_-based ferroelectrics in the recent literature and V:HfO_2_, as depicted in Figure 4d. To provide a reliable comparison given the influence of various parameters, only studies that met the following criteria were considered: cycling frequency ≤1 MHz without recovery breaks and without prewake-up cycling, read frequency ≥1 kHz, final 2*P*_r_ ≥ 10 μC/cm^2^, and homogeneous doping of ferroelectric HfO_2_. Generally, an inverse relationship exists between polarization (2*P*_r_) and endurance, where an increase in the 2*P*_r_ value tends to reduce endurance, while a decrease in 2*P*_r_ tends to enhance it. This relationship, known as the “*P*_r_-endurance dilemma”, underscores the trade-off involved in optimizing ferroelectric materials for both high polarization and long endurance.^16,30,31^ From the endurance tests conducted at varying stimulation electric fields, where breakdown occurred (represented by green empty symbols for V:HfO_2_), a 2*P*_r_ value of 25 μC/cm^2^ was extrapolated to correspond to an endurance of 10^12^ cycles. V:HfO_2_ exhibits robust performance and reliability, achieving relatively large 2*P*_r_ values and high endurance compared with the state of the art.

To examine the temperature stability of the ferroelectric layer, *P*–*V* and *C*–*V* measurements were conducted on similar MFM capacitors at temperatures from 100 to 350 K. As shown in Figure 4e,h, higher temperatures led to increased *E*_c_ and *P*_r_, attributed to injected mobile charges. PUND measurement at 350 K is shown in Figure S6. These tests were performed in a SUSS MicroTec PMC150 cryogenic probe station under vacuum at 10^–5^ mbar.

The impact of annealing temperature was studied by fabricating MFM capacitors at annealing temperatures from 400 to 800 °C, using 2 min RTPs with similar processes. Electrical measurements at 300 K revealed *P*_r_ increased with annealing temperature, reaching 25 μC/cm^2^ at 700 °C, though with larger leakage current (Figure 4f,h). PUND measurement for the 700 °C sample is in Figure S6. Permittivity rose between 450 and 500 °C, then decreased toward 700 °C (Figure 4g). *P*_r_–*E* and ε_r_–*E* curves for 800 °C are not reported due to high leakage currents. No ferroelectricity was observed at 400 °C, but 11.4 μC/cm^2^ remnant polarization was seen at 450 °C. Longer annealing at ∼400 °C needs more investigation. These findings highlight the CMOS compatibility and low thermal budget of V:HfO_2_. A comparison of the annealing and measurement temperatures is summarized in Figure 4h.

Additionally, a thickness study of ferroelectric behavior was performed by fabricating similar capacitors with 8, 16, and 24 nm V:HfO_2_ layers annealed at 600 °C. *P*–*V* and *C*–*V* measurements at RT showed the 16 nm layer had the largest *P*_r_ of 17 μC/cm^2^, while the 8 nm layer exhibited 13.8 μC/cm^2^ with a higher leakage current (Figure 4i). PUND measurement for the 8 nm sample is in Figure S6. Figure 4j–l shows ε_r_–*E* curves from *C*–*V* measurements at frequencies from 10 kHz to 10 MHz. While the 16 nm layer showed greater frequency dispersion, it exhibited the best ferroelectric properties at 10 and 100 kHz. Ferroelectricity and ε_r_ of the 8 and 16 nm layers diminished near 10 MHz. The 24 nm layer exhibited a smaller frequency dispersion, a large ferroelectric response, and relatively high ε_r_ at 10 MHz, suggesting its potential for high-speed memories or high-frequency applications, warranting further investigation at higher frequencies.

A hallmark of ferroelectricity in doped high-*k* dielectrics is the S-shaped polarization-electric field (*P*–*E*) curve, which has been used to demonstrate the so-called negative capacitance (NC) effect. In the Supporting Information (Figure S2), we detail the experimental setup and results of an NC measurements of V:HfO_2_ layer in MFIM stack, showing the expected S-shaped *P*–*E* curve with an equivalent NC of ∼ −540 pF. This confirms that the *P*–*E* plots for V:HfO_2_ closely resemble those of Si:HfO_2_ and HZO, where multiple authors have interpreted this behavior as indicative of the NC effect.^32,33^ These experiments are according to a pulsed method proposed by Hoffmann et al. and Kim et al.^33,34^ and described by inhomogeneous stray energy (ISE) model introduced by Park et al.^35^

In conclusion, we demonstrated V-doped HfO_2_ as a novel CMOS-compatible ferroelectric thin film by fabricating and characterizing multiple V:HfO_2_ samples, identifying 5.9% doping as the optimal level for ferroelectric performance. The versatility of this new ferroelectric extends to compatibility with future VO_2_ Mott insulator devices by a simplified all-ALD process with an identical vanadium precursor. We established that the novel doping with V enables obtaining *P*_r_ of ∼20 μC/cm^2^, *E*_c_ of 1.5 MV/cm, excellent endurance of >10^11^ cycles without failure, and 10-year nonvolatile (NV) retention, competing with leading materials in the field. Material characterization suggests the presence of much larger grain sizes, with respect to other HfO_2_-based ferroelectrics, corroborating the strong piezoresponse detected by PFM. The robust polarization switching, endurance characteristics, and retention properties of the thin film make it an ideal and reliable candidate for nonvolatile memory and neuromorphic devices. Finally, its potential for NC devices is supported by pulsed negative capacitance measurements.

## Supporting Information Available

Further details on the mechanism of observed enhancements due to vanadium doping; detailed explanation of the method used for negative capacitance measurement; supporting figures (PDF)
