# Supplementary material for: Vanadium-Doped Hafnium Oxide: A High-Endurance Ferroelectric Thin Film with Demonstrated Negative Capacitance
Source: Nano Lett. 2025 Feb 7;25(7):2702–8. doi: 10.1021/acs.nanolett.4c05671 (PMC11849039; doi:10.1021/acs.nanolett.4c05671)
Supplement: Supplementary file 1 — nl4c05671_si_001.pdf [file nl4c05671_si_001.pdf]

Supporting Information

Vanadium-Doped Hafnium Oxide: A High-Endurance Ferroelectric Thin Film with Demonstrated Negative Capacitance

Ehsan Ansari,[*,†] Niccolò Martinolli,[†] Emeric Hartmann,[†,‡] Anna Varini,[†] Igor Stolichnov,[†] and Adrian Mihai Ionescu[*,†]

†*Nanoelectronic Device Laboratory, EPFL, Lausanne, 1015, Switzerland*

‡*ENS Paris-Saclay, Gif-sur-Yvette, 91190, France*

E-mail: ehsan.ansari@epfl.ch; adrian.ionescu@epfl.ch

In this document, we include figures and discussions in support of our manuscript. The figures are referenced in the main text and the discussions are aimed at strengthening the validity and understanding of our findings.

Further details on the mechanism of observed enhancements due to vanadium doping:

Vanadium is a multivalent element with four possible valences: +2, +3, +4, and +5, while other reported HfO_2 dopants that induce ferroelectricity exhibit only a single possible valence, such as +2 (e.g., Sr^{2+}), +3 (e.g., Gd^{3+}, La^{3+}, Y^{3+}, Al^{3+}), or +4 (e.g., Si^{4+}, Zr^{4+}). Oxidation states of +3, +4, and +5 have been reported for vanadium as a dopant in chemically synthesized HfO_2 powder, aimed at stabilizing the cubic phase.[1] Our XPS analysis (Figure S1) have also confirmed the dominant presence of V^{3+}, along with a fraction of V^{4+} and a minor fraction of V^{5+}.

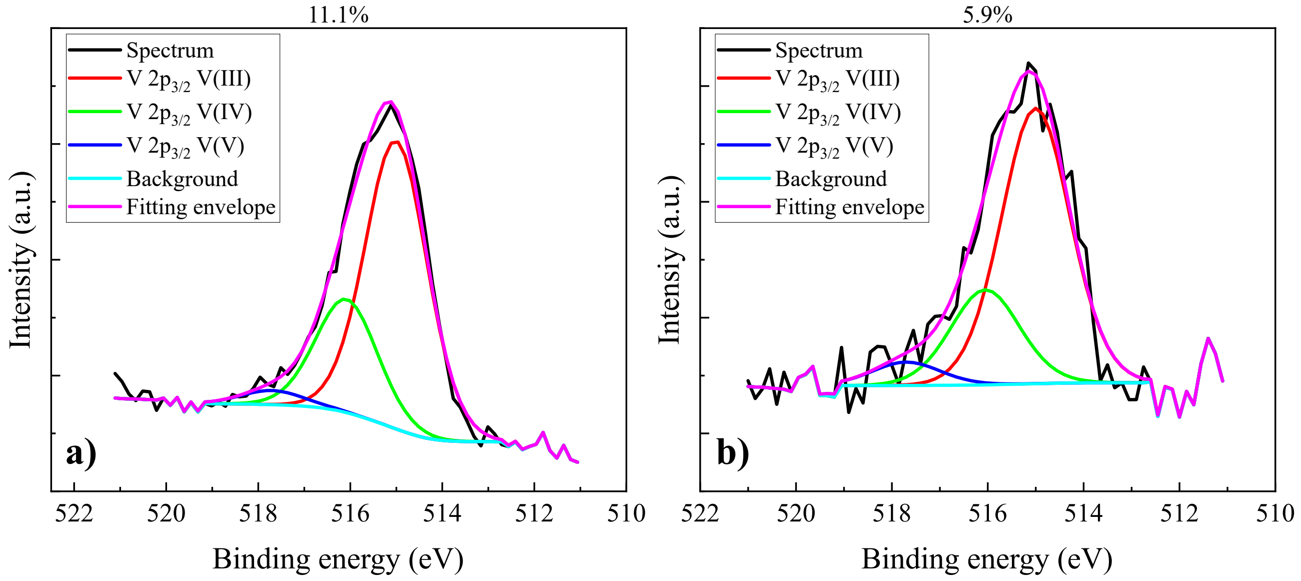

Figure S1: XPS spectra of V 2p in a) 11.1% and b) 5.9% doped V:HfO_2 layers

In addition to the discussion on the strain effect and ionic radii in the manuscript, vanadium doping with a dominant +3 valence state leads to a lower energy of formation for oxygen vacancies,[2] which are one of the key elements in the working mechanism of hafnia-

based ferroelectrics. They appear to be intertwined with polarization switching itself,[3] they have a relatively high diffusion at room temperature, they can transport charge, which leads to leakage, they interact with the electrodes,[4] their diffusion is involved in the wake-up by stabilizing the ferroelectric phase,[5] and they have been demonstrated to be capable of inducing ferroelectricity in HfO_2 with no other factors.[6] Furthermore, oxygen vacancies immobilization and aggregation can bring to the formation of a conductive filament, leading to device failure below breakdown field.[2] The presence of an optimal amount of leakage, which we observe in our samples, is often correlated with higher endurance[7] and we further speculate that the ability of vanadium to switch among its valence states promotes the mobility of oxygen vacancies and the stability of the ferroelectric phase.

Besides, a two-step crystal growth kinetics process has been reported for HfO_2 thin films without a capping layer by Bagmut et al., where orthorhombic or tetragonal phase grains initially grow up to a critical diameter, D^*, before transforming into the monoclinic phase.[8] It is well established that such transformations involve volume expansion and unit cell shearing, which can be suppressed by the presence of a capping layer (e.g., TiN).[9] We hypothesize that vanadium doping lowers the grain boundary energy of the orthorhombic phase relative to the monoclinic phase, further mitigates the shearing strain, and amplifies the kinetic barriers of the transformation into the monoclinic phase. These effects collectively increase the D^* value, resulting in larger ferroelectric grains. However, due to the complex interplay of various factors, including the capping electrode, film thickness, oxygen vacancies, thermal processing, and multivalent doping, further detailed analyses are required to validate this hypothesis. Larger grain sizes and improved crystal quality decrease the density of grain boundaries, which are known sources of charge trapping and scattering. This reduction in grain boundaries enhances polarization switching dynamics, increases remanent polarization, and improves endurance.

Finally, a high breakdown electric field (E_{BD}) of 4.6±0.1 MV/cm was measured across 10 capacitor devices. Considering the endurance cycling experiment shown in Figure 4c,d,

sufficient $2P_r$ values were maintained even under a cycling electric field of 3 MV/cm. Therefore, the high cycling endurance of V:HfO2 can also be attributed to the significant margin between the cycling electric field and E_{BD}. A similar observation has been reported for Ga:HfO_2.[10]

Detailed explanation of the method used for negative capacitance measurement:

Further ferroelectricity analysis was conducted on V:HfO_2 layers through NC measurements in an MFIM stack, using a pulsed method previously proposed by Hoffmann et al., Kim et al.[11,12] and described by inhomogeneous stray energy (ISE) model introduced by Park et al.,[13] to assess their potential for NC applications. Figure S2a illustrates the MFIM capacitor stack with a 3 nm Al_2O_3 layer as a linear dielectric.

Initially, a 50 μs negative voltage pulse was applied to the capacitor to ensure polarization of the ferroelectric layer. Then a Keithley 4200 parameter analyzer was used to apply positive short pulses of ~350 ns with increasing amplitude to the capacitor through a 390 Ω series resistor (R), while an oscilloscope with two channels measured the input voltage and the voltage across the resistor, as shown in Figure S2b. Figure S2c shows the measured input pulse voltage (V_i) versus time. Using the measured voltage of the resistor (V_r) and knowing the resistor value, the current across the circuit loop and the MFIM capacitor (I_c), given by $I_c = V_r/R$, and MFIM capacitor voltage were calculated as a function of time, as shown in Figure S2d,e. By integrating the current response over time, the charge of the MFIM capacitor was extracted as a function of time, as shown in Figure S2f. For each pulse of the increasing pulse train, the maximum stored charge on the capacitor (Q_{max}), residual charge on the capacitor (Q_{res}) when the voltage and current relaxed to zero, and the difference of them ($Q_d = Q_{max} - Q_{res}$) were extracted from Figure S2f and plotted as a function of maximum voltage over the capacitor (V_{c-max}) in Figure S2g.

According to the method reported by Hoffmann et al.,[11] the electric field in the V:HfO_2 layer (E_f) is given by $E_f = (V_{c-max} - Q_d/C_{di})/t_f$, where C_{di} is the capacitance of the dielectric layer and t_f is the thickness of the ferroelectric layer. By experimentally obtaining Q_d and V_{c-max} for each pulse (Figure S2g) and applying the above equation, the electric field E_f was calculated for each pulse. Additionally, the polarization in the ferroelectric layer (P) can be expressed as $P = Q_d/A - \varepsilon_0 E_f + \sigma_{IF}$, where A is the capacitor area, ε_0

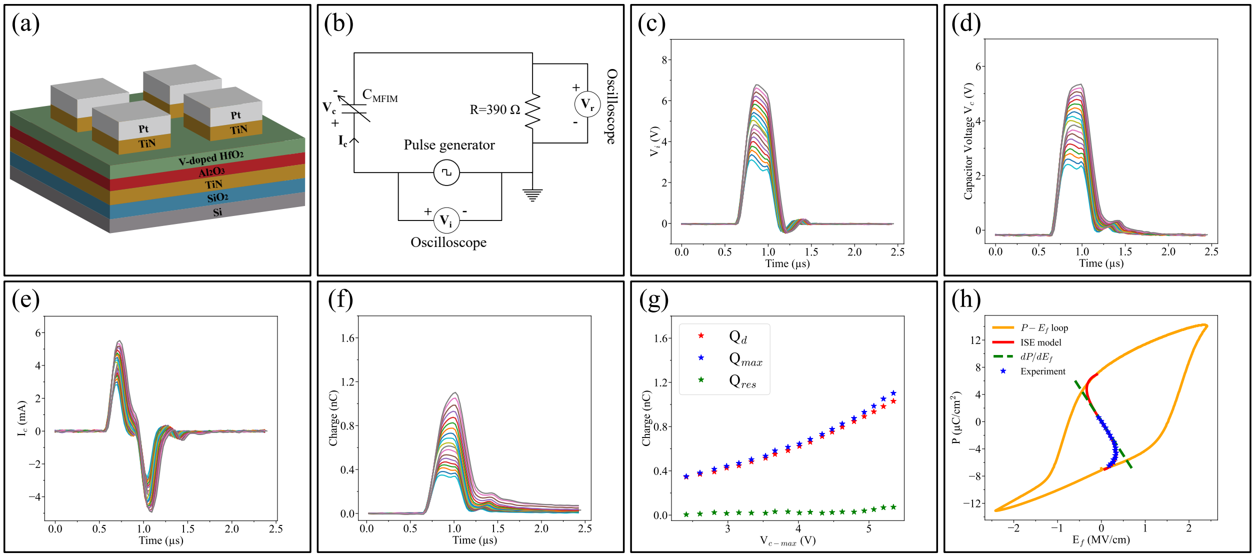

Figure S2: (a) schematic of the MFIM capacitor stack used for the NC measurement, (b) circuit schematic of the NC measurement setup, (c) input pulse voltage, (V_i) and (d) MFIM capacitor voltage, (V_c), as a function of time, (e) MFIM capacitor current and (f) charge as a function of time, (g) MFIM capacitor maximum stored charge (Q_{max}), residual charge (Q_{res}) and the difference of them (Q_d) as a function of maximum voltage over the capacitor, (h) ferroelectric polarization versus electric field. Experimental partial 'S'-shaped characteristics (blue) well matched the ISE Model (red). Amber represents $P - E_f$ curve of an MFM capacitor with 16 nm ferroelectric layer by applying a 10 kHz 3.5 V triangular pulse and green dashed line is the slope of the linear NC region.

is vacuum permittivity and σ_{IF} is an additional charge density at the ferroelectric-dielectric (FE-DE) interface, considered as a shifting constant. Using this equation and the calculated E_f values, the $P-E_f$ curve was plotted, as shown in Figure S2h. A visible partial 'S'-shaped curve was observed, consistent with inhomogeneous stray energy (ISE) quasi-static model for multi-domain structure introduced by Park et al..[13]

This model describes the polarization state of the FE layer through the propagation of domain walls (DW). These DW separate domains with polarization of opposite direction (of width $a1$ and $a2$ respectively). In this model, δ is the variable that describes the proportion of space occupied by each domain orientation ($\delta = (a1 - a2)/(a1 + a2)$). Based on the minimization of free energy density, the ISE model propose the following time-dependent differential equation to describe the evolution of $\delta(t)$:

$$\frac{\partial\delta(t)}{\partial t} = -\frac{2}{T}\mu_{DW}\left[(\frac{C_d}{C_d+C_f})\frac{V_C(t)}{t_f} + (\frac{1}{C_d+C_f})\frac{\lambda P_S\delta(t)}{t_f} + \sum_{n=1}^{\infty}(-1)^n\frac{8\lambda P_S}{\varepsilon_0 k_n^2 T t_f D_n}\sin(\pi n\delta(t))\right] \tag{1}$$

where C_d and C_f are the capacitance density of the dielectric and ferroelectric layer (F/m^2) respectively, t_f is the thickness (m) and P_S is the spontaneous polarization (C/m^2) of the ferroelectric layer respectively, μ_{DW} is the DW mobility ($\mathrm{m^2/s/V}$), T is the domain period (m), $k_n = \frac{2\pi n}{T}$ is the nth wave number (m^{-1}) possible for a domain period T, $D_n = \varepsilon_f * \coth(k_n\frac{t_f}{2}) + \varepsilon_d * \coth(k_n\frac{t_d}{2})$ is the associated coupled dielectric constant, and $0 < \lambda < 1$ is a coefficient describing the reduction of the effective polarization due to charge injected through the DE layer and trapped at the FE-DE interface.

Considering an equivalent circuit of two capacitors (FE and DE) in series with an applied voltage V_C across both, the electric field in the FE layer is given by :

$$E_f = -\left(\frac{C_d}{C_d+C_f}\frac{V_C(t)}{t_f} + \frac{1}{C_d+C_f}\frac{\lambda P_S\delta(t)}{t_f}\right) \tag{2}$$

From equation (1) and (2), the electric field in the FE layer can be approximate in quasi-static state ($\frac{\partial\delta}{\partial t} \approx 0$) by:[13]

$$E_f = \sum_{n=1}^{\infty}(-1)^n\frac{2\lambda P_S T}{\varepsilon_0 n^2\pi^2 t_f D_n}\sin(\frac{\pi n P f}{\lambda P_s}) \tag{3}$$

With $P_f = P_s\delta$, the polarization of the FE layer.

Assuming a quasi-static state for the ferroelectric layer in our experiment and using the parameters $\lambda P_S = 7.15\ \mu C/cm^2$ and $T = 12$ nm, which are consistent with the literature,[14] the $P_f - E_f$ curve of the quasi-static ISE model was calculated and is shown in red (Figure S2h). The ISE model well matched the experimental results, as depicted in Figure S2h. The slope of the green dashed line (dP/dE_f), represents the equivalent relative permittivity

of the linear NC region. Finally, an equivalent capacitance value of $\sim -540\ pF$ was extracted for the ferroelectric using the slope value and equation $C_f = (dP/dE_f) \times (A/t_f)$, which clearly demonstrates the promising potential of V:HfO_2 layer for NC applications.

Supporting figures:

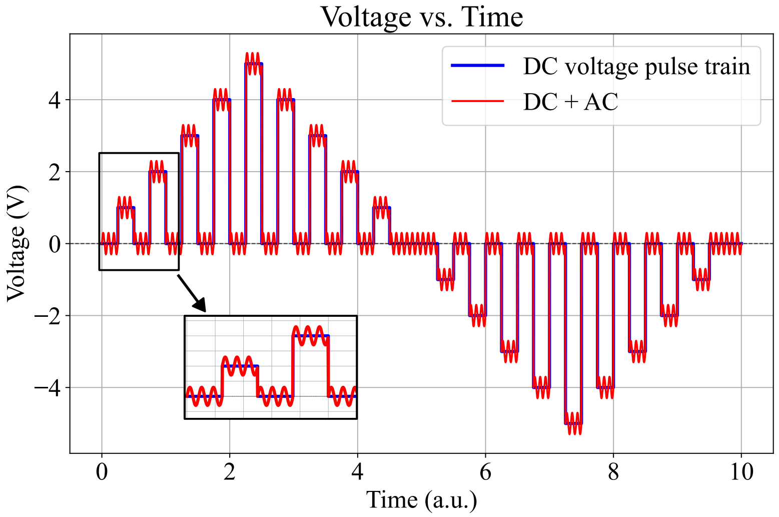

Figure S3: Schematic of the voltage waveform used to acquire PFM loops, consisting of a superposition of 120 DC pulse steps sweeping between 5 v and -5 V, along with an AC signal of 955 kHz with an amplitude of 0.6 V. The small AC signal was consistently applied during the measurement. The on-field loops were acquired while the DC pulse steps were being applied, whereas off-field loops were acquired after each DC pulse step was deactivated.

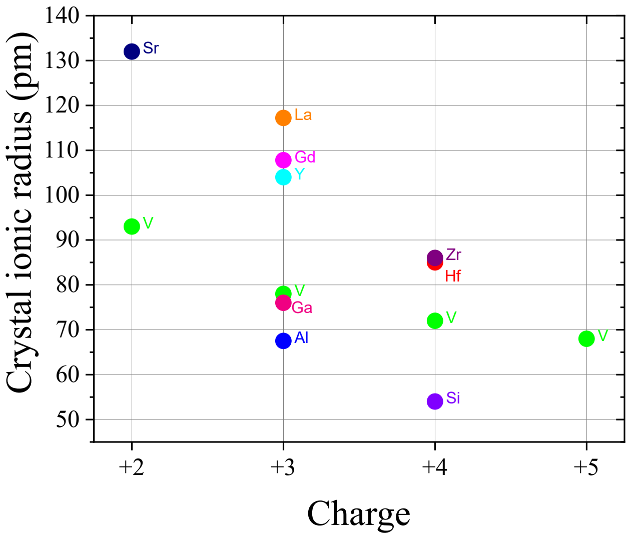

Figure S4: The crystal ionic radii of reported HfO_2 dopants that induce ferroelectricity. The data are taken from Shannon's work.[15]

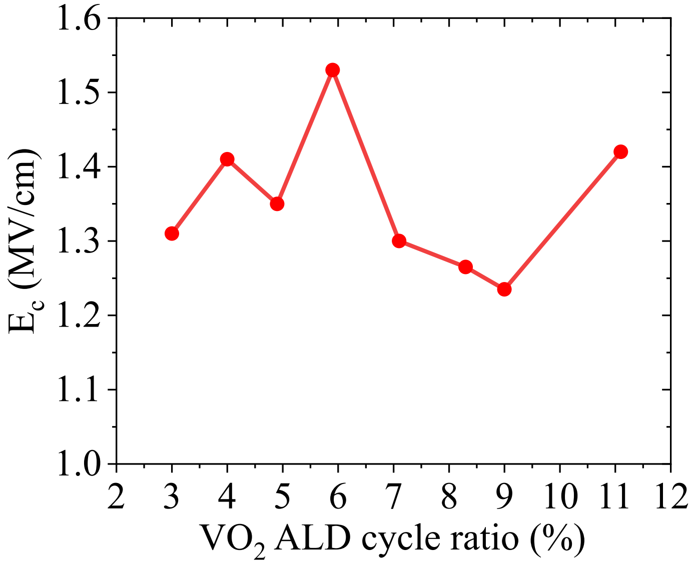

Figure S5: E_c verses VO_2 ALD cycle ratio

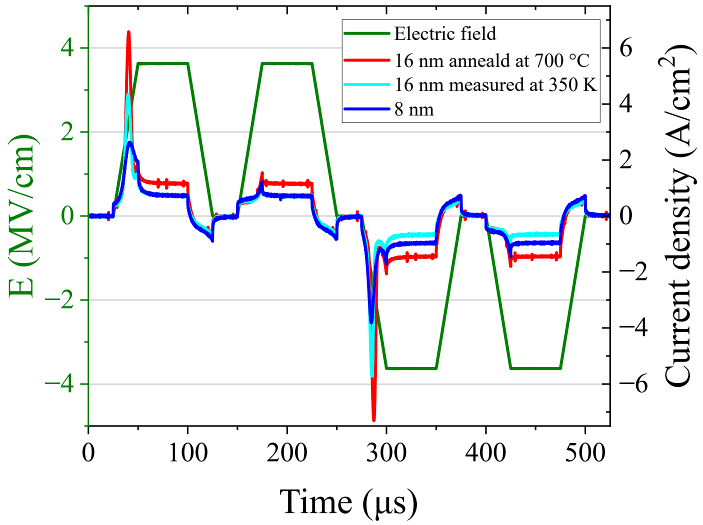

Figure S6: Positive up negative down (PUND) measurement on the 16 nm sample annealed at 700 °C, the 16 nm sample measured at 350 K, and the 8 nm thick sample. Electric field pulse is shown in green

References

(1) Turquat, C.; Leroux, C.; Gloter, A.; Serin, V.; Nihoul, G. V-doped HfO2: thermal stability and vanadium valence. *International Journal of Inorganic Materials* **2001**, *3*, 1025–1032.

(2) Lee, J.; Yang, K.; Kwon, J. Y.; Kim, J. E.; Han, D. I.; Lee, D. H.; Yoon, J. H.; Park, M. H. Role of oxygen vacancies in ferroelectric or resistive switching hafnium oxide. *Nano Convergence* **2023**, *10*, 55.

(3) Noheda, B.; Nukala, P.; Acuautla, M. Lessons from hafnium dioxide-based ferroelectrics. *Nature Materials* **2023**, *22*, 562–569.

(4) Nukala, P.; Ahmadi, M.; Wei, Y.; De Graaf, S.; Stylianidis, E.; Chakrabortty, T.; Matzen, S.; Zandbergen, H. W.; Björling, A.; Mannix, D.; others Reversible oxygen migration and phase transitions in hafnia-based ferroelectric devices. *Science* **2021**, *372*, 630–635.

(5) Starschich, S.; Menzel, S.; Böttger, U. Evidence for oxygen vacancies movement during wake-up in ferroelectric hafnium oxide. *Applied Physics Letters* **2016**, *108*, 032903.

(6) Jaszewski, S. T.; Hoglund, E. R.; Costine, A.; Weber, M. H.; Fields, S. S.; Sales, M. G.; Vaidya, J.; Bellcase, L.; Loughlin, K.; Salanova, A.; others Impact of oxygen content on phase constitution and ferroelectric behavior of hafnium oxide thin films deposited by reactive high-power impulse magnetron sputtering. *Acta Materialia* **2022**, *239*, 118220.

(7) Kim, M.-K.; Kim, I.-J.; Lee, J.-S. Defect Engineering of Hafnia-Based Ferroelectric Materials for High-Endurance Memory Applications. *ACS omega* **2023**, *8*, 18180–18185.

(8) Bagmut, A. G.; Bagmut, I. A. Kinetics of crystals growth under electron-beam crystallization of amorphous films of hafnium dioxide. *Functional Materials* **2018**, *25*, 525–533.

(9) Böscke, T. S.; Müller, J.; Bräuhaus, D.; Schröder, U.; Böttger, U. Ferroelectricity in hafnium oxide thin films. *Applied Physics Letters* **2011**, *99*, 102903.

(10) Huang, T.; Li, Y.-C.; Chen, C.-F.; Li, X.-X.; Gu, Z.-Y.; Zhang, D. W.; Zhu, X.-N.; Lu, H.-L. Demonstration of Robust Breakdown Reliability and Enhanced Endurance in Gallium Doped HfO2 Ferroelectric Thin Films. *IEEE Electron Device Letters* **2023**, *44*, 1476–1479.

(11) Hoffmann, M.; Fengler, F. P. G.; Herzig, M.; Mittmann, T.; Max, B.; Schroeder, U.; Negrea, R.; Lucian, P.; Slesazeck, S.; Mikolajick, T. Unveiling the double-well energy landscape in a ferroelectric layer. *Nature* **2019**, *565*, 464–467, Publisher: Nature Publishing Group.

(12) Kim, Y. J.; Yamada, H.; Moon, T.; Kwon, Y. J.; An, C. H.; Kim, H. J.; Kim, K. D.; Lee, Y. H.; Hyun, S. D.; Park, M. H.; Hwang, C. S. Time-Dependent Negative Capacitance Effects in Al2O3/BaTiO3 Bilayers. *Nano Letters* **2016**, *16*, 4375–4381, Publisher: American Chemical Society.

(13) Park, H. W.; Oh, M.; Hwang, C. S. Negative Capacitance from the Inhomogenous Stray Field in a Ferroelectric–Dielectric Structure. *Advanced Functional Materials* **2022**, *32*, 2200389.

(14) Lee, D. H.; Lee, Y.; Yang, K.; Park, J. Y.; Kim, S. H.; Reddy, P. R. S.; Materano, M.; Mulaosmanovic, H.; Mikolajick, T.; Jones, J. L.; Schroeder, U.; Park, M. H. Domains and domain dynamics in fluorite-structured ferroelectrics. *Applied Physics Reviews* **2021**, *8*, 021312.

(15) Shannon, R. D. Revised effective ionic radii and systematic studies of interatomic distances in halides and chalcogenides. *Acta Crystallographica Section A: Crystal Physics, Diffraction, Theoretical and General Crystallography* **1976**, *32*, 751–767, Publisher: International Union of Crystallography.
